# Strong decline in female sterilization rates in Norway after the introduction of a new copayment system: a registry based study

**DOI:** 10.1186/1472-6874-7-12

**Published:** 2007-08-27

**Authors:** Inger J Bakken, Finn E Skjeldestad, Unni Schøyen, Marit G Husby

**Affiliations:** 1 Department of Epidemiology, SINTEF Health Research, N-7465 Trondheim, Norway; 2 Norwegian University of Science and Technology (NTNU), N-7491 Trondheim, Norway

## Abstract

**Background:**

January 1, 2002, copayment for outpatient female sterilization in Norwegian public hospitals increased from 33 euros to 750 euros after a revision of the health care system. The aim of the present study was to investigate the effect of the new copayment system on female sterilization epidemiology.

**Methods:**

We retrieved data on all female sterilizations 1999–2005 (N = 23 1333) from the Norwegian Patient Register, an administrative register to which it is mandatory for all hospitals to report. Sterilizations with diagnostic codes indicative of vaginal delivery, caesarean section, spontaneous abortion, ectopic pregnancy, and termination of pregnancy were analyzed separately. All other sterilizations were defined as "interval sterilization".

**Results:**

An abrupt fall in female sterilization was observed after the raise in copayment. Age-adjusted incidence rates dropped from 6.3–6.8 per 1000 women in 1999–2001 to 2.2–2.3 per 1000 women during 2002–2005. Interval sterilizations dropped to 25% of the previous level after the rise in copayment while sterilizations in conjunction with caesarean section and postpartum sterilization remained constant.

**Conclusion:**

For many Norwegian women seeking contraception, sterilization is no longer an available alternative.

## Background

A new copayment system for sterilization was introduced in Norway January 1, 2002. Copayment for outpatients increased from 33 euros to 750 euros for women and from 27 euros to 156 euros for men. No copayment is charged from inpatients. This substantial change in copayment for sterilization was part of a revision of the health care system [1]. Norwegian citizens have the right to free medical care within certain limitations. In January 2002, a system was commenced where the right to medical care was divided into three levels, with sterilization in the lowest priority group with corresponding high copayment (50% and 100% of estimated costs for women and men, respectively).

The Norwegian Patient Register is an administrative database containing activity data for all public and private hospitals in Norway [2]. Reporting is mandatory and is linked to the re-imbursement system for funding of health services.

The sudden change in patient copayment together with the complete record in the Patient Register presented us with a unique opportunity to study the impact of the price of the procedure on the incidence of female sterilization.

## Methods

The study was approved by the Norwegian Data Inspectorate. Authorization for the use of sensitive health data was obtained from the Norwegian Ministry of Health and Social affairs.

The Norwegian Patient Register comprises more than 20 variables, including basic administrative data (institution, dates of admission and discharge), patient data (sex, year of birth, county of residency), and medical data. Medical data include up to eight diagnoses coded according to the International Classification of Diseases system, 10^th ^Revision (ICD-10) and up to ten surgical procedures coded according to NOMESKO's Classification of Surgical Procedures (NCSP).

In Norway, sterilization can be demanded by women 25 years and older. We identified women 25–49 years old who had undergone sterilization as indicated by diagnosis code Z302 ("sterilization") and/or a procedure code in the LGA group ("female sterilization").

Sterilizations with diagnostic codes indicative of vaginal delivery (ICD-10 codes starting with O8, except O82 and O84.2), caesarean section (codes starting with O82 and code O84.2), spontaneous abortion (codes starting with O0, except codes starting with O00 and O04), ectopic pregnancy (codes starting with O00), and termination of pregnancy (codes starting with O04) were analyzed separately. All other sterilizations were defined as "interval sterilization".

Sterilization procedures are also reported to Statistics Norway as individual case report forms [3]. We retrieved data from this source for comparison.

Incidence was calculated as the number of female sterilizations relative to the number of same-aged women per year. Age was categorized into five-year age-groups. Age adjusted incidence rates were calculated by using the direct method with the female population 25–49 years old in Norway 1999 as the reference. Population figures were provided by Statistics Norway.

SPSS for Windows, version 13.0 (SPSS, Chicago, IL) were used in all analyses.

## Results

We identified 23315 women with a diagnostic code and/or a procedural code indicative of sterilization over the years 1999–2005. We excluded 131 women younger than 25 years and 51 women older than 49 years. The total study population comprised 23133 women aged 25–49 years.

Most study participants (20931/23133, 90.5%) were identified with both a diagnostic and a procedural code for sterilization. Mean age at sterilization was 37.1 years (SD 4.9), with only minor variations over the study years (data not shown).

The number of female sterilizations was relatively stable during 1999–2001, fell abruptly in 2002, and remained thereafter at a constant low level (Table 1). During the years 1999–2003 the number of female sterilizations in Statistics Norway corresponded to 77–90% of the numbers in the Norwegian Patient Register. Age adjusted incidence rates fell from 6.3–6.8 per 1000 women during 1999–2001 to 2.2–2.3 per 1000 in 2002–2005. During the entire period, incidence rates for sterilization peaked in the 35–39 year age group (Table 1). The decrease in incidence rates over the study period was lowest among the youngest women and strongest in the oldest age group (53% and 73%, respectively).

**Table 1:** Total number of sterilizations from the Norwegian Patient Register and Statistics Norway^1^, and incidence of sterilization per 1000 same-aged women^2^

	1999	2000	2001	2002	2003	2004	2005
N (Norwegian Patient Register)	5250	5080	5483	1888	1794	1859	1779
N (Statistics Norway)	4727	4481	4625	1455	1580	-	-
25–29	1.6	1.7	1.8	0.8	0.9	0.8	0.8
30–34	6.8	6.7	6.5	2.8	2.7	2.8	2.8
35–39	12.9	12.1	13.3	4.5	4.1	4.1	3.8
40–44	9.7	9.4	10.5	2.9	2.7	3.0	2.8
45–49	1.9	1.7	1.9	0.5	0.5	0.5	0.5

25–49 (age adjusted)^3^	6.6	6.3	6.8	2.3	2.2	2.3	2.2

^1^Data from one county was lacking in 2002

^2^Based on data from the Patient Register

^3^Adjusted to the age distribution of women 25–49 years old in Norway 1999

The fall in sterilizations from 2001 to 2002 was confined to interval sterilizations (Table 2). In this group, the average annual number of sterilizations during 2002–2005 was 79% lower than the average number during 1999–2001. The number of sterilizations in conjunction with caesarean section and postpartum sterilizations remained stable throughout the study period (inpatients), while sterilizations carried out in conjunction with pregnancy termination decreased (mainly an outpatient procedure).

**Table 2:** Number of female sterilizations (% of total) undertaken at interval or in conjunction with pregnancy, data from the Norwegian Patient Register 1999–2005

	1999	2000	2001	2002	2003	2004	2005
Interval	4421 (84.2)	4253 (83.7)	4627 (84.4)	1157 (61.3)	1032 (57.5)	1115 (60.0)	1043 (58.6)
Caesarean section	415 (7.9)	416 (8.2)	459 (8.4)	501 (26.5)	524 (29.2)	532 (28.6)	534 (30.0)
Vaginal delivery	163 (3.1)	134 (2.6)	116 (2.1)	114 (6.0)	99 (5.5)	107 (5.8)	121 (6.8)
Termination of pregnancy	197 (3.8)	221 (4.4)	212 (3.9)	74 (3.9)	86 (4.8)	72 (3.9)	52 (2.9)
Ectopic pregnancy	34 (0.6)	34 (0.7)	41 (0.7)	29 (1.5)	39 (2.2)	24 (1.3)	18 (1.0)
Spontaneous abortion	20 (0.4)	22 (0.4)	28 (0.5)	13 (0.7)	14 (0.8)	9 (0.5)	11 (0.6)

Total	5250	5080	5483	1888	1794	1859	1779

## Discussion

In the present study, we observed a strong decline in female sterilizations when copayment increased from 33 euros to 750 euros January 1, 2002. The decrease was restricted to interval sterilizations.

The major strength of present study is that we had access to data on all female sterilizations carried out in Norway from a single data source. The Patient Register contains large amounts of information, including demographic data and medical data on diagnoses and procedures. Comparison with data from Statistics Norway showed a lower number of female sterilizations in the latter database for all years 1999–2003, indicating higher completeness in reporting to the Patient Register. A detailed comparison of sterilizations from the Patient Register and from Statistics Norway can be found elsewhere [4].

The abrupt fall in sterilization rates which was restricted to interval sterilization clearly coincided with the introduction of the new copayment system and we are certain that this fall was directly linked to the increase in copayment. Data from Statistics Norway showed a declining trend in female sterilization during 1985–1994, a period without changes in the copayment system [3]. This declining trend was however gradual, and during the years most recent to the introduction of the new copayment system (1995–2001), the annual number of sterilizations as reported by Statistics Norway was stable.

The situation in Norway with a strong sudden increase in price of sterilization with a corresponding abrupt fall in rates is to the best of our knowledge unique. Gradually declining trends and low sterilization levels have however been observed in other countries. Thus, it cannot be excluded that the sterilization rates would have declined in Norway also without changes in the copayment system. We observed sterilization rates slightly above 6 per 1000 same-aged women before the introduction of the new payment system and somewhat higher than 2 per 1000 thereafter. In Great Britain, sterilization rates declined from 5 per 1000 women in 1992 to 3.5 per 1000 in 1999 [5]. During 1981 to 1995, sterilization rates fell from 15 per 1000 women to 6 per 1000 in New South Wales, Australia [6]. A comparative study of sterilization in the Nordic countries showed large country-wise variations in the years 1980, 1988, 1990 and 1994 [7]. While rates in Denmark were low and relatively stable (approximately 3 per 1000 women), a declining rate was observed in Sweden (from 6 per 1000 to 4 per 1000). In Finland female sterilization rates peaked at 10 per 1000 in 1990, and fell thereafter to 8 per 1000 in 1995 [7]. National statistics from Finland show a gradual decline in sterilization from 1996, to 5 per 1000 women in 2005 [8].

In the Nordic countries [7] and in most other countries [9] male sterilization rates have traditionally been lower than female rates. It could have been expected that the introduction of a differential copayment for male and female sterilization, with a considerably lower cost for men, might have been reflected in an increase in the number male sterilizations. However, the annual number of male sterilizations in Norway remained stable throughout all years 1996–2003 [3].

Norwegian inpatients are exempted from copayment. We observed that five in six female sterilizations were interval sterilizations before the rise in copayment. While interval sterilizations dropped to 25% of the level in previous years after the rise in copayment, the numbers of sterilizations in conjunction with caesarean section and postpartum sterilization remained constant (inpatients). Interval sterilizations comprised approximately 60% of all sterilizations 2001 and onwards, comparable to figures from the US showing that 50% of sterilizations were performed at interval during 1994–96 [10].

## Conclusion

This population-based register study shows that after the introduction of a 750 EUR copayment many Norwegian women no longer consider sterilization as an available contraception method.

## Competing interests

The author(s) declare that they have no competing interests.

## Authors' contributions

IJB was the lead author for the paper, retrieved the data, and contributed to study design, data analysis, and data interpretation. US and MGH contributed to study design, data analysis, and data interpretation. FES obtained funding and contributed to study design, data interpretation, and critical revision of the manuscript. All authors contributed to the write up and have read and approved the final manuscript.

## Pre-publication history

The pre-publication history for this paper can be accessed here:


